# Whole-transcriptome dynamics reveal distinct regulatory networks during sugar beet vernalization

**DOI:** 10.3389/fpls.2026.1942667

**Published:** 2026-09-11

**Authors:** Xixuan Zhou, Zhengzhi Zhong, Liumin Wang, Yufei Ku, Zedong Wu, Zhi Pi

**Affiliations:** 1 Academy of Modern Agriculture and Ecological Environment, Heilongjiang University, Harbin, China; 2 Key Laboratory of Sugar Beet Genetic Breeding, Heilongjiang University, Harbin, China

**Keywords:** *Beta vulgaris*, flowering time, long non-coding RNA, microRNA, vernalization

## Abstract

Sugar beet (*Beta vulgaris* L.) is a biennial crop that requires prolonged cold exposure to initiate flowering—a process known as vernalization. This cold requirement restricts the growing season and limits cultivation options. However, the molecular networks that distinguish vernalization from general cold stress remain poorly characterized in this crop. Here, we performed a time-course whole-transcriptome analysis across five vernalization stages (0, 2, 6, 10, and 14 weeks) and identified 3,598 mRNAs that were specifically altered only after sufficient cold exposure. Many of these encode histone modifiers (including enzymes involved in histone phosphorylation, methylation, and acetylation) and transcription factors. Their expression patterns suggest a chromatin-based regulatory mechanism, although this inference is derived from transcript abundance rather than direct chromatin measurements. In contrast to the *FLC*-centered pathway in *Arabidopsis*, sugar beet may employ a putatively distinct regulatory logic: long non-coding RNAs (lncRNAs) such as MSTRG.54931.1 and MSTRG.64283.2 are predicted to preferentially associate with stress-related kinases rather than floral repressors, and microRNAs (miRNAs) predominantly respond at early cold stages, with limited contribution to later vernalization memory. These findings suggest a new regulatory framework for vernalization in a non-model crop and may provide a foundation for breeding bolting-tolerant varieties.

## Introduction

In temperate climates, many plants require an extended period of winter cold to initiate flowering in spring—a process termed vernalization. This developmental switch ensures that reproduction occurs under favorable conditions and is a major determinant of growing season and crop yield (Bastow et al., 2004). In *Arabidopsis thaliana*, the molecular basis of vernalization has been dissected in detail: the floral repressor *FLC* is epigenetically silenced through histone modifications (H3K27me3, H3K9me2) mediated by Polycomb repressive complexes, and this silenced state is maintained after the plants are returned to warm conditions (Sung and Amasino, 2004; Searle, 2006). Non-coding RNAs, including the long non-coding RNAs *COOLAIR*, *COLDAIR*, and *COLDWRAP*, play critical roles in this process by guiding chromatin remodeling at the *FLC* locus (Heo and Sung, 2011; Csorba et al., 2014; Hung et al., 2022). These histone modifications have been directly validated through chromatin immunoprecipitation (ChIP) assays in *Arabidopsis*, whereas the present study focuses on transcriptomic correlates of histone modifier gene expression. In recent years, non-coding RNAs have been increasingly implicated in flowering regulation across diverse plant species. In *Arabidopsis*, lncRNAs such as *COOLAIR*, *COLDAIR*, and *COLDWRAP* have been shown to regulate FLC expression through epigenetic mechanisms (Heo and Sung, 2011; Csorba et al., 2014; Hung et al., 2022). In Chinese cabbage, a comprehensive transcriptomic analysis identified over 1,500 lncRNAs involved in plumule-vernalization, with 280 ceRNA pairs participating in the vernalization response (Dai et al., 2023). In wheat, the miR172-APETALA2-like module integrates vernalization and plant age signals to control flowering time (Debernardi et al., 2022). In sugar beet, however, the roles of ncRNAs—particularly lncRNAs and miRNAs—in vernalization have not been systematically investigated.

In contrast, the vernalization mechanism in sugar beet (*Beta vulgaris* L.) remains poorly understood. Sugar beet is a biennial crop that accumulates sucrose in its taproot during vegetative growth. Premature bolting—the transition to reproduction—depletes these reserves and reduces root yield by 10–20% and sugar content by 0.8–1.6% (Biancardi, 2005). While the *BvBTC1–BvFT1–BvFT2* module has been identified as a key regulator of bolting (Pin et al., 2010; Dally et al., 2014; Kuroda et al., 2026), several important questions remain unanswered. First, unlike *Arabidopsis*, the *FLC* ortholog *BvFL1* does not appear to be the major effector of vernalization in beet (Vogt et al., 2014). However, we acknowledge that *BvFL1* may play an accessory role, and its exclusion from our core regulatory network is based on the previous functional characterization rather than on new expression data from this study. We also recognize that Vogt et al. studied biennial beet germplasm, whereas our material (KWS9147) is a bolting-resistant cultivar; the functional role of *BvFL1* may differ across genetic backgrounds, and thus the biennial conclusion should be extrapolated to our system with caution. Second, the roles of non-coding RNAs—particularly lncRNAs and miRNAs—in beet vernalization have not been systematically investigated. Third, histone modification dynamics during beet vernalization remain to be investigated.

To address these gaps, we performed a whole-transcriptome analysis of sugar beet leaves across a vernalization time course (0, 2, 6, 10, and 14 weeks). Our aims were to: identify transcripts that are specifically associated with the attainment of the fully vernalized state; characterize the involvement of histone modifiers, transcription factors, lncRNAs, and miRNAs; and determine whether sugar beet uses a regulatory logic similar to or distinct from the *Arabidopsis* model. Our results suggest a previously unrecognized regulatory network in which lncRNAs converge on stress-related kinases rather than floral repressors, and miRNAs serve mainly as early cold responders. These findings may provide a new framework for understanding vernalization in sugar beet and offer potential targets for breeding bolting-resistant varieties.

## Materials and methods

### Plant material and vernalization treatment

Seeds of the bolting-resistant sugar beet variety KWS9147 were sown in square pots (12 cm × 12 cm × 10 cm) and grown in a greenhouse at 24 ± 2 °C with a 16-h light/8-h dark photoperiod at 200 μmol m^−2^ s^−1^ light intensity. At 24 days after sowing, seedlings were transferred to a cold chamber at 4 ± 2 °C under the same light conditions for vernalization. At 0, 2, 6, 10, and 14 weeks of vernalization, young leaves were harvested and stored at −80 °C for RNA extraction. The 2-week time point was chosen as the earliest sampling time based on preliminary observations that short-term cold exposure (1–7 days) in sugar beet primarily induces general cold stress responses rather than vernalization-specific transcriptional changes. While we acknowledge that the absence of 1-day and 3-day cold treatments limits our ability to fully distinguish cold acclimation from vernalization progression, the focus of this study was on long-term cold exposure (2–14 weeks) because vernalization in biennial sugar beet requires at least 10 weeks of chilling to achieve bolting competency (Pi et al., 2020). The vernalization stages were determined based on bolting-related agronomic traits (bolting index, bolting delay, and leaf number at bolting) observed during the 16-week chilling treatment, as previously described (Pi et al., 2020). Accordingly, five representative time points (0, 2, 6, 10, and 14 weeks) were selected for transcriptome sequencing to capture the pre-vernalization, transitional, and fully vernalized stages (**Supplementary Table 1**).

### RNA sequencing and transcriptome assembly

Total RNA was extracted using TRIzol reagent (Invitrogen, USA), treated with TURBO DNase I (Ambion, USA), and quality-verified by Nanodrop and agarose gel electrophoresis. Strand-specific RNA-seq libraries were prepared using the NEB Next Ultra Directional RNA Library Prep Kit and sequenced on the Illumina HiSeq platform. Clean reads were filtered using a Perl script to remove low-quality reads, adapters, and poly-N sequences. Reads were mapped to the RefBeet-1.2 reference genome using StringTie (Pertea et al., 2015). Transcripts longer than 200 nt were assessed for protein-coding potential using CPC2, CNCI, CPAT, and Pfam (Kang et al., 2017; Sun et al., 2013; Wang et al., 2013; Mistry et al., 2021). Novel mRNAs and lncRNAs were classified based on length, exon number, and coding potential.

### miRNA sequencing and analysis

Small RNA libraries were prepared using the TruSeq™ Small RNA Sample Prep Kit (Illumina) and sequenced on the Illumina platform. Clean reads were filtered for length (18–30 nt) and aligned to the miRBase database and the sugar beet reference genome using miRDeep2 (Friedländer et al., 2012). miRNAs were quantified using TPM, and differentially expressed miRNAs (DE-miRNAs) were identified using DESeq2 (Love et al., 2014). Target genes were predicted using psRNATarget.

### Differential expression and network analysis

mRNAs, lncRNAs, and miRNAs with a fold change > 2 and an adjusted *P*-value < 0.05 (Benjamini–Hochberg correction) were considered differentially expressed. Only transcripts with an average expression > 1 in at least one sample were retained for further analysis. Data are presented as mean ± SD from three biological replicates. Statistical significance between groups was determined using Student’s *t*-test. To identify nuclear-encoded DE-mRNAs, the subcellular localization of proteins encoded by the 3,598 vernalization-associated DE-mRNAs was predicted using DeepLoc (Almagro Armenteros et al., 2017) with default parameters. DeepLoc is a deep learning-based tool that predicts protein subcellular localization from amino acid sequences. Transcripts encoding proteins predicted to be localized to the nucleus were selected for further analysis. This resulted in a set of 446 nuclear-encoded DE-mRNAs, which were subsequently subjected to GO enrichment analysis. GO enrichment analysis was performed using AgriGO (Tian et al., 2017) with the PLAZA 4.0 dicot database as reference. Transcription factors were identified using PlantTFDB (Jin et al., 2017). Cis- and trans-regulatory targets of lncRNAs were predicted using LncTar and co-expression analysis (Spearman correlation > 0.95, *P* < 0.05). Networks were visualized with Cytoscape (Shannon et al., 2003).

### qRT-PCR validation

To validate the transcriptome sequencing results, SYBR Green-based quantitative real-time PCR (qRT-PCR) was performed. Total RNA was extracted from ground leaf tissues using RNA-easy isolation reagent (Vazyme, Nanjing, China) following the manufacturer’s protocol, which included lysis, precipitation, washing, and dissolution steps. RNA concentration and purity were determined using a NanoDrop 2000c spectrophotometer (Thermo Fisher Scientific, USA) by measuring the OD260/280 ratio, and RNA integrity was assessed via agarose gel electrophoresis. For each sample, 1 μg of total RNA was treated with gDNA Eraser to remove genomic DNA contamination and then reverse-transcribed into cDNA using the PrimeScript RT reagent Kit with gDNA Eraser (Takara, Dalian, China) according to the manufacturer’s instructions. The resulting cDNA was diluted 3-fold with Easy Dilution (Takara, Dalian, China) prior to qRT-PCR analysis. Gene-specific primers were designed using the Primer-BLAST program (NCBI). All primer pairs were designed to span at least one exon and were verified to produce no non-specific amplification in the sugar beet genome. The primer sequences used in this study are listed in **Supplementary Table 2**.

Three DE-mRNAs (*BvRNP1*, *BvHDT1*, *BvFVE1*) and three DE-lncRNAs (MSTRG.29583.1, MSTRG.9690.1, MSTRG.38394.1) were selected for qRT-PCR validation. qRT-PCR was performed using the SYBR Premix Ex Taq™ II kit (Takara, Dalian, China) on a CFX-96 Real-time System (Bio-Rad, USA) with a two-step amplification protocol: 95 °C for 30 s for initial denaturation, followed by 40 cycles of 95 °C for 5 s and 60 °C for 34 s for annealing and extension. After the amplification cycles, a melting curve analysis was conducted by heating from 60 °C to 95 °C at a ramp rate of 0.5 °C per 5 s to verify product specificity. The sugar beet *BvGAPDH* gene (Bv5_110740_ygnn) was used as an internal reference control. Relative expression levels were calculated using the 2^−ΔΔCt^ method (Livak and Schmittgen, 2001), and statistical significance was determined using one-way ANOVA with a threshold of P < 0.05. Correlation between qRT-PCR and RNA-seq data was assessed using Pearson’s correlation coefficient.

## Results

### Transcriptomic reprogramming during vernalization

To identify transcripts associated with vernalization, we performed RNA-seq on sugar beet leaves at 0, 2, 6, 10, and 14 weeks of cold exposure. A total of 261.09 Gb of clean data was generated, with 52.4 to 73.8 million clean reads per sample. GC content, Q20, and Q30 were calculated from the clean data, and mapping efficiency ranged from 73.68% to 79.93%. In total, 7,586 DE-mRNAs were identified across all time points.

**Figure 1:**
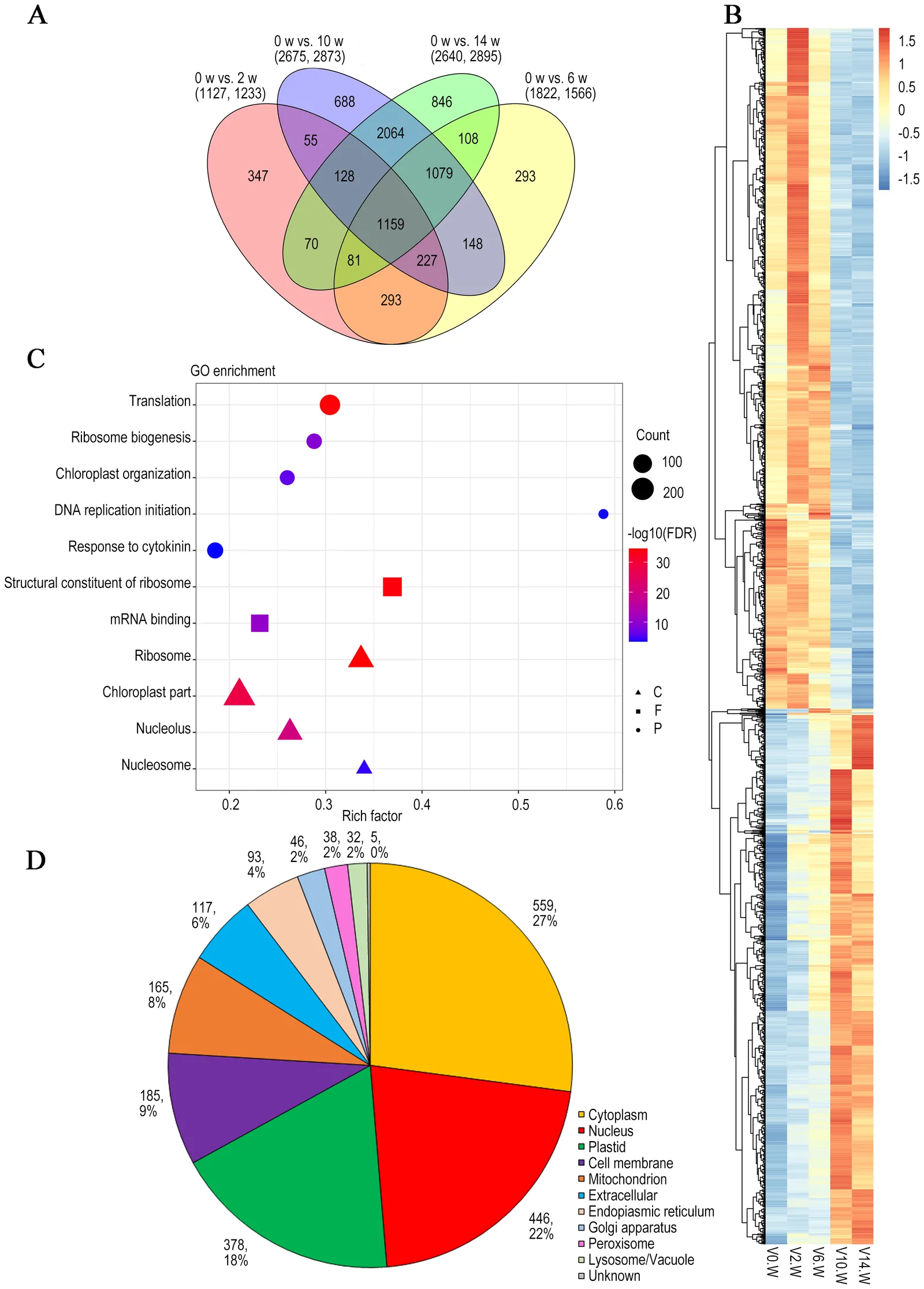
The expression pattern and GO enrichment analysis of DE-mRNA after vernalization in sugar beet. **(A)** Venn diagram of DE-mRNA. **(B)** Hierarchical clustering of DE-mRNAs identified at 2, 6, 10, and 14 weeks of vernalization (relative to 0-week control). **(C)** GO enrichment of DE-mRNA identified in both 10 w and 14 w treatment. **(D)** Subcellular localization classification of DE-mRNA-encoded proteins. This Venn diagram is based on the transcriptome dataset previously published in our earlier study (Zhang et al., 2024), but is reanalyzed here to highlight transcripts specifically associated with the lateresponding vernalization stages.

Compared to the 0-week control, 2,360, 3,388, 5,548, and 5,535 DE-mRNAs were detected at 2, 6, 10, and 14 weeks, respectively (**Figure 1A**). Notably, 3,598 mRNAs showed significant changes exclusively at the fully vernalized stages (10 and 14 weeks), with 2,064 of these altered at both time points. These transcripts are here referred to as “late-responding” or “prolonged cold-associated” mRNAs, rather than strictly “vernalization-specific,” as we cannot exclude the possibility that some of these transcripts may represent sustained cold stress responses rather than vernalization memory per se.Hierarchical clustering revealed that among these 2,064 mRNAs, 908 were upregulated and 1,153 were downregulated at both 10 and 14 weeks (**Figure 1B**). GO enrichment analysis of these late-responding mRNAs showed significant enrichment for transcriptional regulation, translation, and nuclear-localized proteins (**Figures 1C, D**). Subcellular localization prediction indicated that 446 of these mRNAs encode nuclear proteins, suggesting a potential chromatin-based regulatory mechanism.

### Histone modifiers and transcription factors

**Figure 2:**
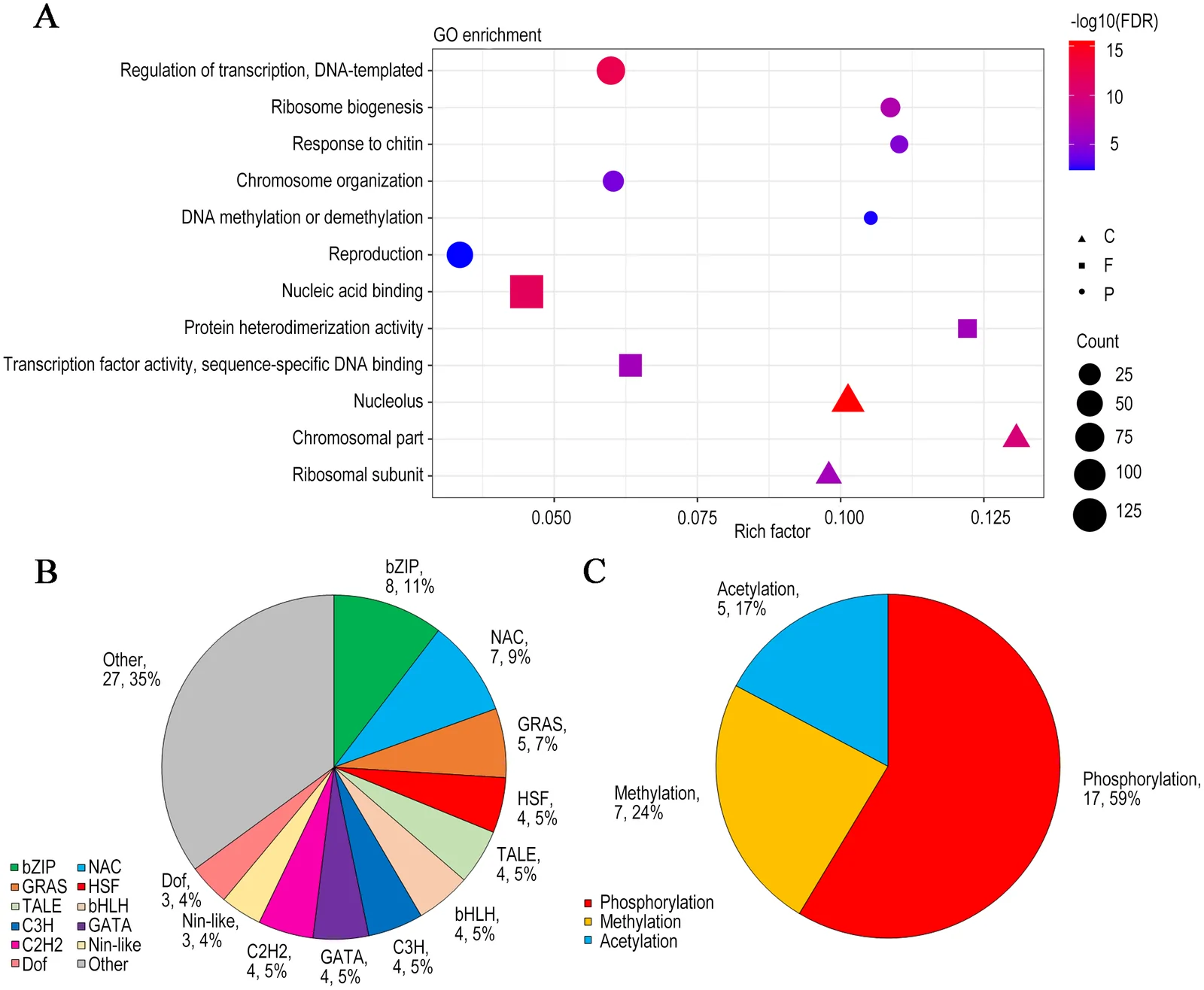
Functional annotation of nuclear-encoded DE-mRNAs. **(A)** GO enrichment. **(B)** Key transcription factors. **(C)** protein-modifying enzymes.

Among the 446 nuclear-encoded DE-mRNAs, GO analysis revealed enrichment for chromatin organization, transcriptional regulation, and DNA methylation (**Figure 2A**). We identified 77 transcription factors from 29 families, including bZIP, NAC, GRAS, and a CO-like factor (Bv9_208530_ycgs) previously associated with flowering (**Figure 2B**). Additionally, we identified multiple genes involved in histone phosphorylation, methylation, and acetylation (**Figure 2C****;** **Table 1**). These include kinases (BvHaspin, BvAurora2, BvATM), methyltransferases (BvATX1, BvEZA1, BvATXR6), demethylases (BvJMJ18, BvJMJ706, BvLDL1), and deacetylases (BvHDT1, BvHDT2). The coordinated regulation of these chromatin modifiers suggests that histone modifications may play a key role in establishing the vernalized state.

**Table 1:** Protein-modifying enzymes involved in vernalization.

Gene name	Entry ID	Description	Modification	Fold change*	Histone site**
*BvHaspin*	Bv4_079570_xazj	serine/threonine-protein kinase haspin	Phosphorylation	-1.86; -2.19	H3T3ph, H3T11ph
*BvAurora2*	Bv2_032910_fdft	serine/threonine-protein kinase Aurora-2	Phosphorylation	-2.04; -4.70	H3S10ph
*BvATM*	Bv5_117140_ujaw	serine/threonine-protein kinase ATM isoform X3	Phosphorylation	1.61; 1.23	H2AS139ph
*BvMAPKKK1*	Bv2_032410_iqnp	mitogen-activated protein kinase kinase kinase 1	Phosphorylation	1.68; 1.57	—
*BvYODA*	Bv6_134510_ftsu	mitogen-activated protein kinase kinase kinase YODA	Phosphorylation	1.80; 1.46	—
*BvPP2C56*	Bv8_197440_jnqo	protein phosphatase 2C 56-like	Dephosphorylation	2.54; 1.91	—
*BvATX1*	Bv1_007710_sigj	histone-lysine N-methyltransferase ATX1-like isoform X1	Methylation	-1.09; -1.41	H3K4me3
*BvEZA1*	Bv5_121850_runy	histone-lysine N-methyltransferase EZA1 isoform X2	Methylation	2.70; 2.44	H3K27me3
*BvATXR6*	Bv3_051490_hzom	histone-lysine N-methyltransferase ATXR6	Methylation	-2.89; -3.30	H3K27me1
*BvLDL1*	Bv9_215320_xtxg	lysine-specific histone demethylase 1 homolog 1	Demethylation	-1.06; -1.13	H3K4me3
*BvJMJ18*	Bv4_097100_oxei	lysine-specific demethylase JMJ18	Demethylation	1.78; 1.64	H3K4me3
*BvJMJ706*	Bv5_106970_xzta	lysine-specific demethylase JMJ706	Demethylation	-1.19; -1.22	H3K9me3
*BvHDT1*	Bv9_211080_qkgc	histone deacetylase HDT1-like	Deacetylation	-1.68; -1.88	H3K9ac
*BvHDT2*	Bv9_211090_esde	histone deacetylase HDT2	Deacetylation	-1.57; -1.64	—

*Fold change values at 10 and 14 weeks of vernalization, respectively.

**Histone sites are predicted based on the functional characterization of homologous genes in *Arabidopsis thaliana*; direct experimental validation in sugar beet has not been performed in this study.

### Transcription factor binding networks

To identify upstream regulators of the nuclear-encoded DE-mRNAs, we predicted transcription factor binding sites in their promoter regions. We identified 39 differentially expressed transcription factors that bind to the promoters of 125 nuclear-encoded DE-mRNAs, forming 163 regulatory pairs with correlation coefficients > 0.8. These included MYB, ERF, GATA, C3H, and TCP family members (the full network is shown in **Supplementary Figure 1**). Notably, the ERF transcription factor (Bv3_052580_wurf) was associated with histone modification-related genes, while a MYB factor (Bv2_031880_ghua) bound to promoters of genes encoding cell cycle and histone organization proteins.

### Long non-coding RNA networks

**Figure 3:**
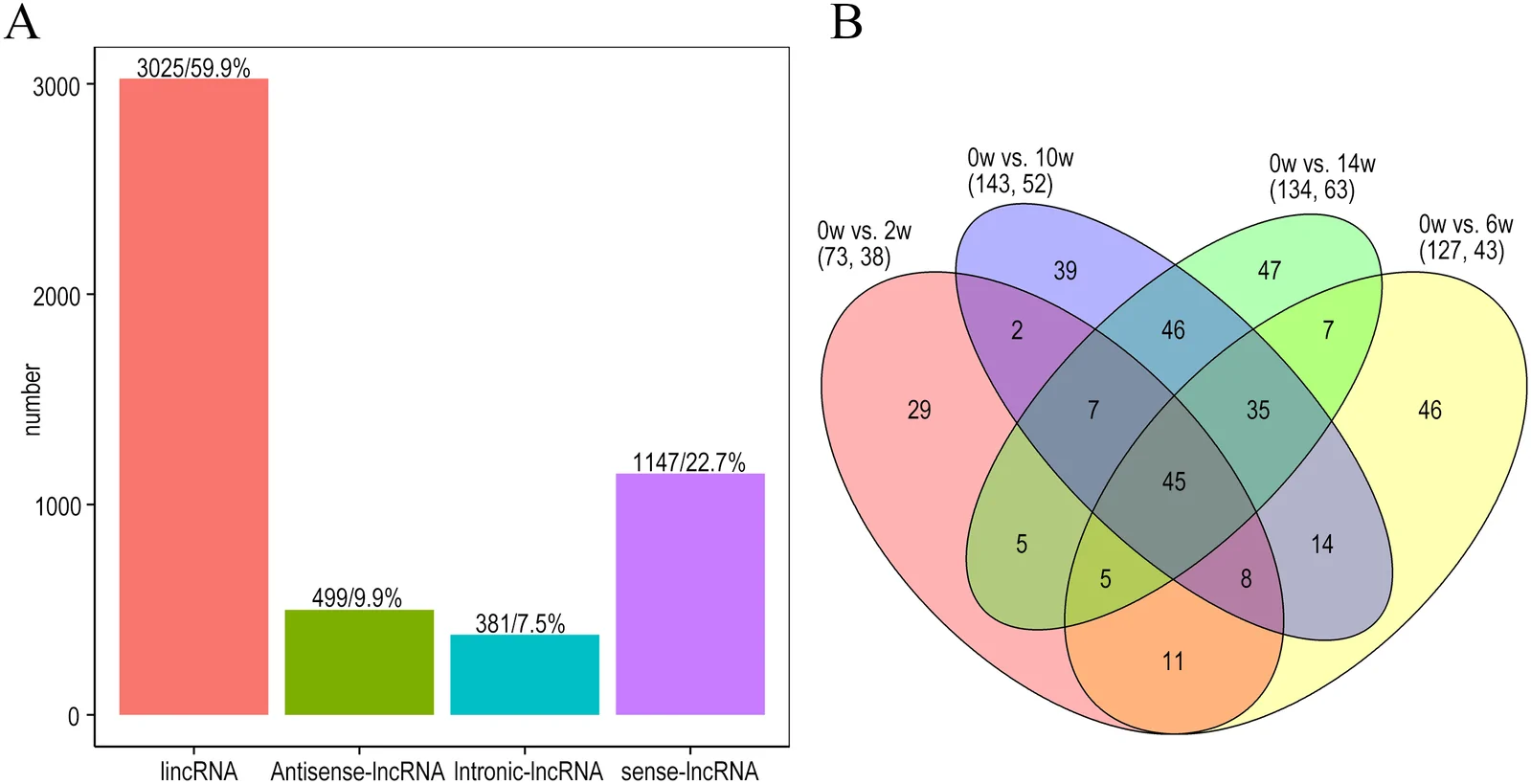
DE-lncRNAs involved in vernalization. **(A)** Classification. **(B)** Venn diagram.

We identified 5,052 lncRNAs, including 3,025 intergenic (59.9%), 499 antisense (9.9%), 381 intronic (7.5%), and 1,147 sense (22.7%) lncRNAs (**Figure 3A**). Among these, 345 were differentially expressed during vernalization (DE-lncRNAs). Of these, 132 showed significant changes only at 10 or 14 weeks, and 46 were altered at both fully vernalized time points (**Figure 3B**).

To identify hub lncRNAs that may integrate vernalization signals, we constructed regulatory networks between DE-lncRNAs and DE-mRNAs encoding protein-modifying enzymes and transcription factors. Network centrality analysis identified four hub lncRNAs: MSTRG.54931.1, MSTRG.10638.11, MSTRG.64283.2, and MSTRG.4864.1. Three of these (MSTRG.54931.1, MSTRG.10638.11, MSTRG.64283.2) showed significant expression changes only at 10 and 14 weeks, suggesting dose-dependent regulation with vernalization duration.

Specifically, MSTRG.54931.1 and MSTRG.10638.11 were co-expressed with kinases involved in cold signaling and stress responses, including BvMAPKKK1 (a mitogen-activated protein kinase kinase kinase involved in cold and brassinosteroid signaling), BvYODA (a MAPKKK regulating cell proliferation), and BvPP2C56 (a protein phosphatase implicated in ABA signaling and stress adaptation). MSTRG.64283.2 trans-regulated multiple histone-modifying enzymes, including BvATM (histone H2A kinase), BvATX1 (H3K4 methyltransferase), and BvHDT1 (H3K9 deacetylase), suggesting its potential involvement in chromatin remodeling during vernalization. Additionally, MSTRG.4864.1 and MSTRG.64283.2 showed potential regulatory relationships with multiple transcription factors, including ERF, MYB, and TCP family members, which are known to regulate stress-responsive and developmental genes.

**Figure 4:**
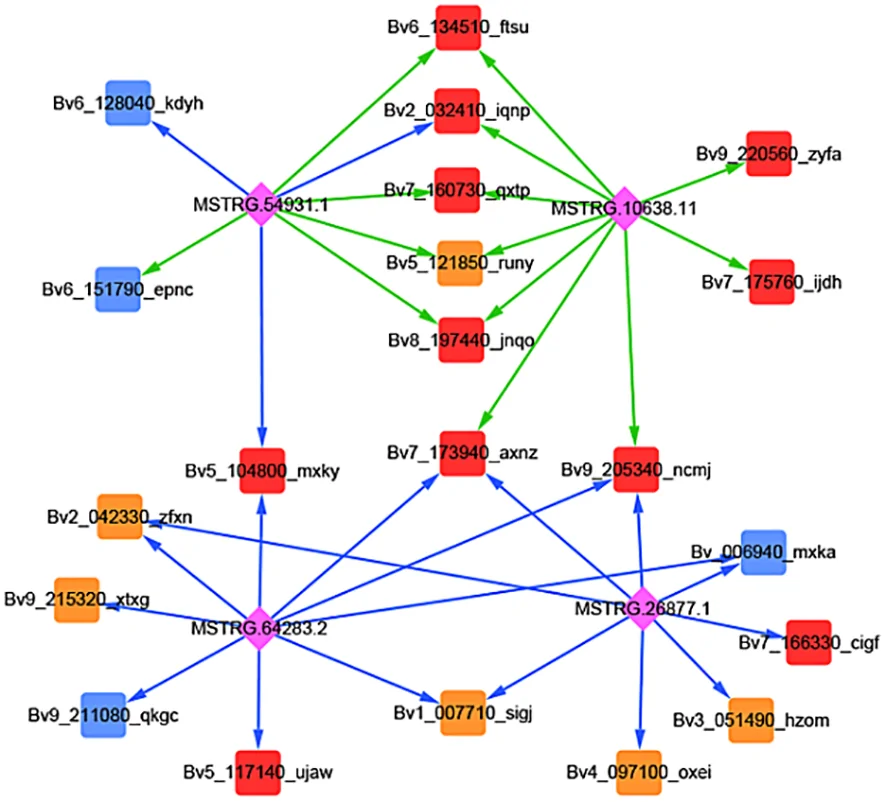
Predicted lncRNA–mRNA regulatory network for protein-modifying enzymes. Regulatory relationships are based on Spearman correlation (r > 0.95) and LncTar target prediction, and should be considered as testable hypotheses rather than validated interactions.

**Figure 5:**
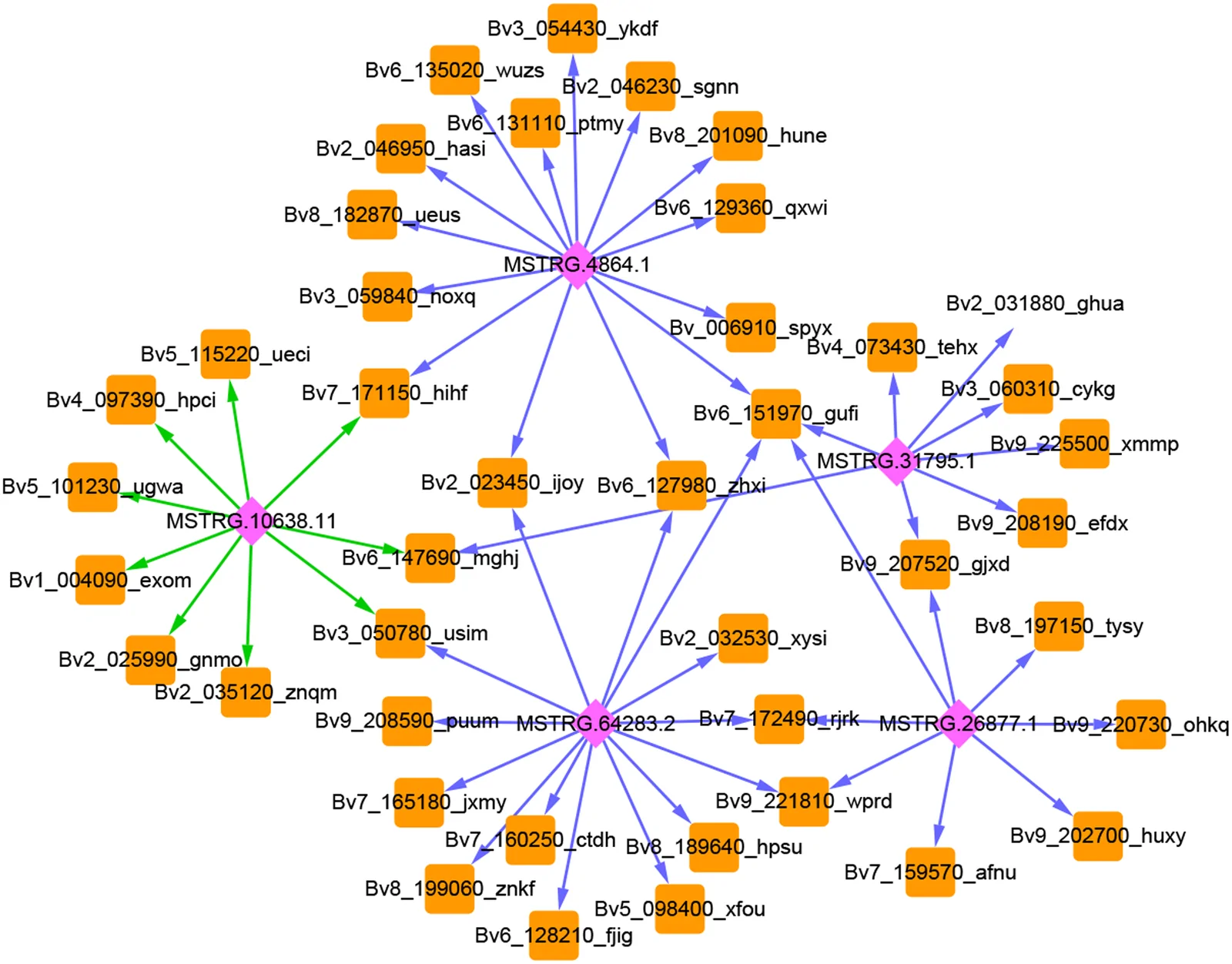
Predicted lncRNA–mRNA regulatory network for transcription factors. Regulatory relationships are based on Spearman correlation (r > 0.95) and LncTar target prediction, and should be considered as testable hypotheses rather than validated interactions.

The regulatory networks of these hub lncRNAs with protein-modifying enzymes and transcription factors are shown in (**Figures 4**, **5**), respectively.

### MicroRNA dynamics

**Figure 6:**
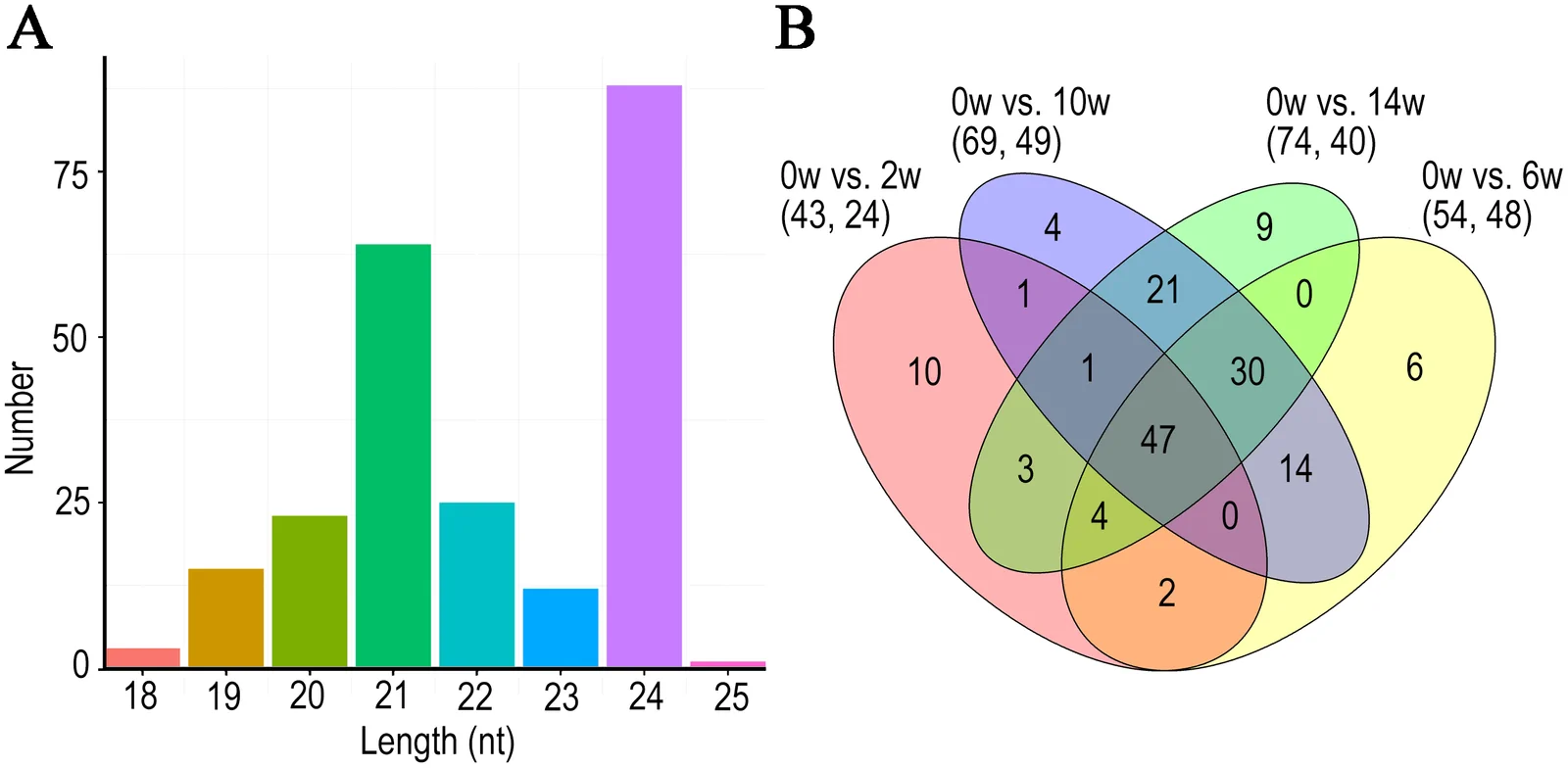
DE-miRNAs involved in vernalization. **(A)** Length distribution of identified miRNAs. The X-axis represents miRNA length in nucleotides (nt), and the Y-axis represents the number of miRNAs identified at each length. **(B)** Venn diagram showing the number of DE-miRNAs detected at 2, 6, 10, and 14 weeks of vernalization compared to the 0-week control.

Small RNA sequencing identified 237 miRNAs, of which six were known miRNAs. The length distribution was mainly centered at 21 nt and 24 nt (**Figure 6A**). In total, 151 DE-miRNAs were detected, with 34 showing significant changes only at 10 or 14 weeks, and 21 at both fully vernalized stages (**Figure 6B**).

Target prediction revealed that seven miRNAs may bind to mRNAs involved in histone methylation, and nine miRNAs may target phosphorylation-related genes. However, unlike lncRNAs, most DE-miRNAs exhibited significant changes primarily at 2 weeks of vernalization, with limited changes at later time points. Among the DE-miRNAs, only novel-miR172 showed consistent changes at both 10 and 14 weeks. This miRNA was predicted to target *BvCDC1*, which may be associated with cell division. Nin-like and HD-Zip transcription factors were predicted to be regulated by multiple DE-miRNAs. Full miRNA regulatory networks are shown in **Supplementary Figure 2** (protein-modifying enzymes) and **Supplementary Figure 3** (transcription factors).

### Validation of transcriptome data

**Figure 7:**
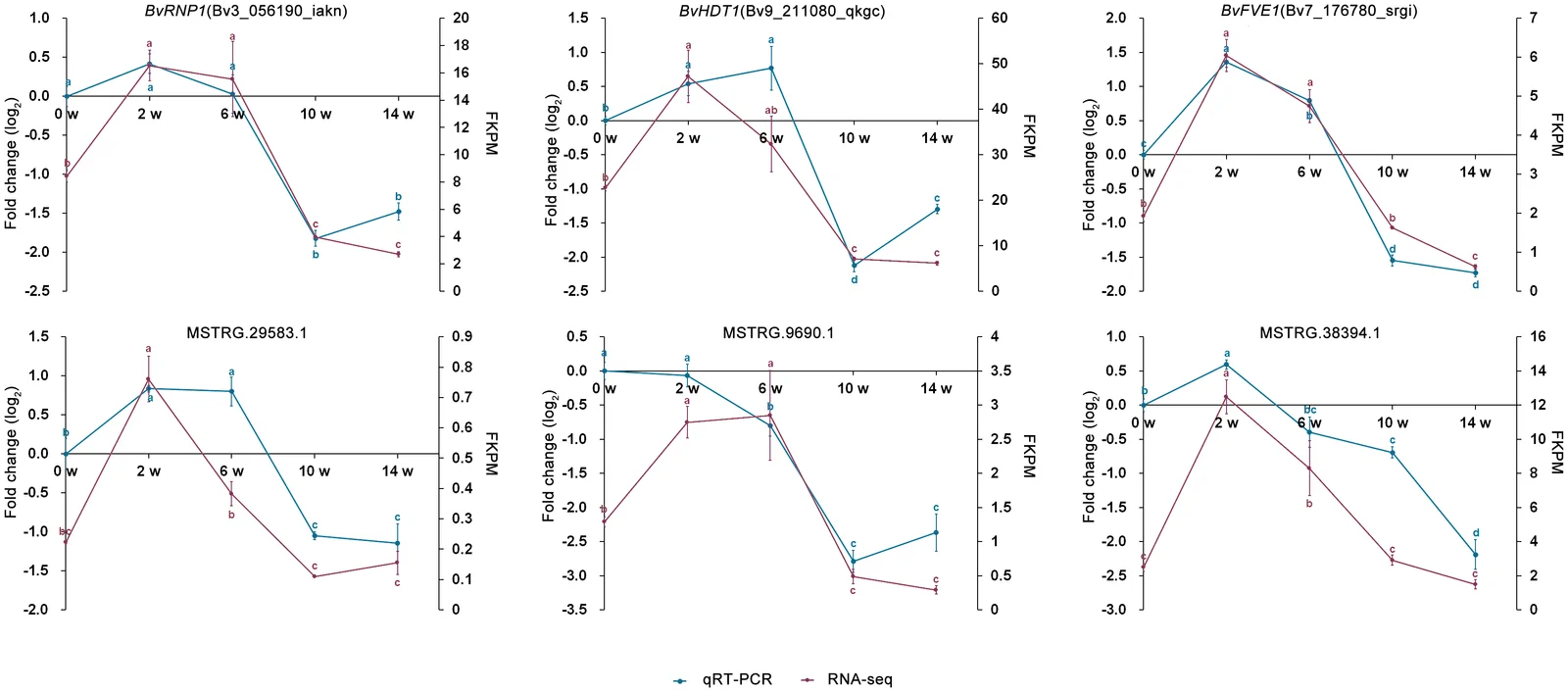
qRT-PCR validation of selected DE-mRNAs (*BvRNP1, BvHDT1, BvFVE1*) and DE-lncRNAs (MSTRG.29583.1, MSTRG.9690.1, MSTRG.38394.1). The X-axis represents vernalization time points (0, 2, 6, 10, and 14 weeks). The left Y-axis shows the fold change (log_2_-transformed) for qRT-PCR data (blue lines), and the right Y-axis shows FPKM values for RNA-seq data (red lines).

We validated the RNA-seq data by qRT-PCR analysis of three DE-mRNAs (*BvRNP1*, *BvHDT1*, *BvFVE1*) and three DE-lncRNAs (MSTRG.29583.1, MSTRG.9690.1, MSTRG.38394.1). All six transcripts showed expression trends consistent with the RNA-seq data, with correlation coefficients of 0.894, 0.880, and 0.929 for the three mRNAs, and 0.821, 0.747, and 0.699 for the three lncRNAs (**Figure 7**). These results confirm the accuracy and reliability of our transcriptomic analyses.

## Discussion

Vernalization sensitivity is a key determinant of the sugar beet growing season, but the underlying regulatory networks have remained obscure compared with the well-characterized *Arabidopsis* model. In this study, we performed a time-course whole-transcriptome analysis and identified a potentially distinct regulatory architecture in sugar beet that appears to differ from the canonical FLC-centric pathway. This study is designed as a discovery-oriented transcriptomic survey to generate testable hypotheses for future functional studies, rather than as a mechanistic validation of individual regulatory interactions.

We found that 3,598 mRNAs are specifically altered only after sufficient cold exposure (10–14 weeks). These include multiple histone-modifying enzymes—kinases, methyltransferases, demethylases, and deacetylases—that target histones H2A, H3, and H4. This observation is consistent with the idea that chromatin remodeling is a conserved feature of vernalization across species. However, unlike *Arabidopsis*, where H3K27me3 deposition at *FLC* is the central event, sugar beet may employ a broader repertoire of histone modifications, including phosphorylation, methylation, and acetylation (**Table 1**). Whether these modifications act in a coordinated or sequential manner to establish vernalization memory remains to be determined, but our data provide a comprehensive starting point for such investigations. To assess whether the identified histone modifier genes exhibit coordinated expression dynamics, we examined their temporal expression profiles across the five vernalization time points. Notably, histone methyltransferase genes (*BvATX1, BvEZA1, BvATXR6*) and demethylase genes (*BvJMJ18, BvJMJ706, BvLDL1*) showed distinct but partially overlapping temporal patterns, with the methyltransferases generally downregulated during prolonged cold exposure and the demethylases showing divergent responses. Whether these histone modifier genes target overlapping sets of chromatin loci remains to be determined, as genome-wide histone modification profiling (e.g., ChIP-seq) would be required to map their genomic targets.

We identified 77 transcription factors from 29 families, including bZIP, NAC, and GRAS. Notably, *BvCO-like* (Bv9_208530_ycgs) was significantly expressed after sufficient vernalization, whereas its close relative *BvCO* showed no significant changes. (Dally et al., 2018) speculated that multiple *BvCO-like* genes regulate *BvFT1* and *BvFT2* expression. Our results are consistent with this hypothesis but also highlight functional divergence within the *CO* family. This divergence may reflect a regulatory network that has been reshaped by the specific life history of sugar beet as a biennial crop.

In *Arabidopsis*, lncRNAs such as *COOLAIR*, *COLDAIR*, and *COLDWRAP* function together with histone modifiers to epigenetically silence the floral repressor *FLC* (Heo and Sung, 2011; Csorba et al., 2014; Hung et al., 2022). In sugar beet, the *FLC* ortholog *BvFL1* does not play a key role in vernalization (Vogt et al., 2014). Nevertheless, our findings indicate that lncRNAs in sugar beet also cooperate with chromatin-modifying machinery: the hub lncRNAs we identified (MSTRG.54931.1, MSTRG.10638.11, MSTRG.64283.2) were predicted to associate with histone-modifying enzymes (*BvATM*, *BvATX1*, *BvHDT1*) and stress-related kinases (*BvMAPKKK1*, *BvYODA*, *BvPP2C56*). This suggests that a common regulatory logic—lncRNA-mediated coordination with chromatin modifiers—may operate across species, but the specific lncRNA molecules and their target genes are not conserved. This may reflect a more ancestral or alternative strategy for linking cold perception to developmental reprogramming, one that has diverged substantially between *Arabidopsis* and sugar beet. The association of lncRNAs with stress-related kinases also suggests potential crosstalk between cold perception and hormone signaling pathways. Recent reviews have highlighted how auxin signaling integrates environmental stress responses with developmental plasticity (Ali et al., 2025), and it is plausible that similar integration occurs during vernalization in sugar beet, where cold stress signals may be transduced through kinase cascades to modulate flowering transition.

Unlike lncRNAs, most DE-miRNAs exhibited significant changes primarily at 2 weeks of vernalization, with limited changes at 10 and 14 weeks. This temporal partitioning suggests that miRNAs are mainly involved in early cold acclimation rather than in sustaining the vernalized state. Similar patterns have been observed in wheat, where miR172 integrates vernalization and age signals, but its main regulatory impact occurs early in the cold response (Debernardi et al., 2022). The difference in miR172 expression dynamics between sugar beet and wheat is noteworthy. In wheat, miR172 maintains elevated expression throughout vernalization, whereas in sugar beet, novel-miR172 showed only limited late-stage expression. This discrepancy may reflect species-specific differences in the regulatory architecture of vernalization: in wheat, miR172 functions as an integrator of both vernalization and age pathways, whereas in sugar beet, the vernalization response appears to be more heavily dependent on lncRNA-mediated chromatin modulation. Alternatively, the limited late-stage expression of novel-miR172 in sugar beet could indicate that its primary role is in early cold acclimation rather than in vernalization memory, consistent with our observation that most DE-miRNAs peaked at 2 weeks of cold exposure. Importantly, the predicted targeting of *BvCDC1* by novel-miR172 requires experimental validation (e.g., via 5′ RLM-RACE or transient co-expression assays), and we therefore consider this as a testable hypothesis rather than a confirmed regulatory interaction. In sugar beet, only novel-miR172 showed consistent changes at both fully vernalized stages, potentially regulating *BvCDC1* and cell division. The miR156-SPL module, which is conserved in many species including beet (Asgari et al., 2022), was not prominently represented among our late-stage DE-miRNAs, further suggesting that miRNAs may contribute less to vernalization memory than lncRNAs and histone modifications.

Based on these findings, we propose a working model for vernalization in sugar beet. The following model integrates our experimentally observed transcriptomic changes (including differential expression of histone modifiers, lncRNAs, and miRNAs) with computationally predicted regulatory interactions. Components based solely on correlation and network prediction should be considered as testable hypotheses requiring functional validation. Cold exposure triggers early miRNA-mediated stress responses and transient transcriptional changes. As vernalization progresses, specific lncRNAs accumulate in a dose-dependent manner, recruiting or modulating chromatin-modifying complexes (kinases, methyltransferases, deacetylases) that reshape the epigenetic landscape. This lncRNA-mediated chromatin remodeling, rather than a single *FLC*-like switch, may ultimately establish the vernalized state. The involvement of stress-related kinases suggests that cold signaling pathways may be directly coupled to developmental reprogramming, a feature that may be more prominent in biennials than in rapid-cycling annuals. In this model, the experimentally supported components include the time-dependent accumulation of specific lncRNAs (MSTRG.54931.1, MSTRG.64283.2) and the differential expression of histone modifier genes. The regulatory relationships between these lncRNAs and their predicted targets, however, remain hypothetical until validated by functional assays such as CRISPR-Cas9-mediated knockouts or RNA interference.

Several limitations should be acknowledged. Our conclusions are based on transcriptomic data and computational predictions; protein-level changes and direct epigenetic marks (e.g., ChIP-qPCR) have not been examined. We acknowledge that the absence of post-rewarming time points in our experimental design limits our ability to distinguish between genes involved in cold acclimation and those truly mediating vernalization memory. Future studies incorporating rewarming time courses (e.g., 1–4 weeks after return to 25 °C) will be required to define the “vernalization-specific” transcriptome more precisely, as has been done in *Arabidopsis* where the maintenance of FLC silencing after cold exposure is the hallmark of vernalization memory. The lack of very short-term cold treatment time points (e.g., 1–3 days) means that some of the early transcriptional changes observed at 2 weeks may reflect cold stress responses rather than vernalization-specific programming. Nevertheless, our primary focus was on identifying transcripts associated with the acquisition of the fully vernalized state (10–14 weeks), which is the period when bolting competency is achieved. The regulatory roles of the identified lncRNAs and miRNAs require functional validation through genetic perturbation—for example, CRISPR-Cas9 or virus-induced gene silencing in sugar beet. In addition, while our transcriptomic data identified multiple histone modifier genes that are differentially expressed during vernalization, we cannot determine from expression data alone which specific histone modifications are functionally required for establishing the vernalized state. Determining functional necessity would require targeted approaches such as ChIP-seq to map the genomic distribution of specific histone marks, combined with genetic perturbation of individual histone modifier genes. Additionally, we used only one genotype (KWS9147), and the regulatory networks may vary among cultivars. Whether the networks identified here are conserved among sugar beet cultivars with contrasting bolting sensitivity remains an open question that will require comparative transcriptomic analyses across multiple genotypes. The role of *BvFL1* in sugar beet vernalization remains to be fully resolved. While Vogt et al. (2014) demonstrated that *BvFL1* is not a major regulator, our study cannot rule out a minor or accessory role for this gene. Future studies combining *BvFL1* expression analysis with functional perturbation (e.g., overexpression or knockout) will be required to clarify its contribution, if any, to vernalization regulation in sugar beet. Despite these limitations, our study provides a comprehensive resource of candidate genes and regulatory interactions that can now be tested experimentally. Beyond bulk transcriptomics, emerging single-cell and spatial transcriptomic approaches have the potential to resolve cell-type-specific responses to vernalization across different tissues and developmental stages (Ali et al., 2026). Applying such cellular-resolution methods to sugar beet vernalization could uncover whether the lncRNA-mediated regulatory networks identified here operate specifically in the shoot apical meristem or other cell populations, and how cell-to-cell communication contributes to the establishment of vernalization memory.

Future studies using CRISPR-Cas9-mediated knockout of hub lncRNAs, chromatin immunoprecipitation (ChIP) for histone marks, and single-cell transcriptomics will be required to validate the regulatory roles proposed here and to establish causality between lncRNA expression, chromatin remodeling, and flowering transition.

## Conclusion

In summary, we propose a distinct regulatory network for vernalization in sugar beet. Unlike the *FLC*-centric paradigm in *Arabidopsis*, sugar beet may employ lncRNA-mediated circuits that converge on stress-related kinases and chromatin modifiers, with miRNAs primarily serving early cold responses. This temporal and functional partitioning of regulatory layers may provide a new framework for understanding how biennial plants perceive and remember winter cold. Our findings also identify potential targets for breeding bolting-resistant sugar beet varieties, enabling early spring sowing and extended autumn harvesting to improve sugar production.

## Data Availability

The raw RNA‑sequencing data generated in this study have been deposited in the NCBI Sequence Read Archive and are publicly available under accession number PRJNA773644. The miRNA sequencing data have also been deposited in NCBI under accession number PRJNA1495426 and are publicly available.
