# Supplementary material for: Whole-transcriptome dynamics reveal distinct regulatory networks during sugar beet vernalization
Source: Front Plant Sci. 2026 Sep 11;17:1942667. doi: 10.3389/fpls.2026.1942667 (PMC13612567; doi:10.3389/fpls.2026.1942667)
Supplement: Supplementary file 1 [file Supplementaryfile1.docx]

Supplementary Material

# 1 Supplementary Tables

**Supplementary Table S1** Bolting-related agronomic traits of sugar beet (KWS9147) during vernalization

| **Weeks of chilling** | **Bolting Index (BI, %)** | **Bolting Delay (BD, days)** | **Leaf Number at Bolting (LNB)** | **Stage** |
| --- | --- | --- | --- | --- |
| 0 | 0 | — | — | I |
| 2 | 0 | — | — | I |
| 4 | 0 | — | — | I |
| 6 | 0 | — | — | I |
| 8 | 0 | — | — | I |
| 10 | 66.7 | 38.5 | N.S. | II |
| 12 | 79.2 | 29.8 ± 3.4 | N.S. | II |
| 14 | 95.8 | 20.2 ± 2.8 | N.S. | III |
| 16 | 100 | 15.3 ± 2.5 | N.S. | III |

Stage I: Insufficient vernalization (no bolting); Stage II: Transitional period (partial bolting); Stage III: Sufficient vernalization (nearly all bolted). Data are presented as mean ± SD from three biological replicates. BD: days from transfer to 25°C to visible bolting initiation. LNB: number of leaves at bolting. LNB showed no significant difference among vernalized treatments; values shown are approximate means based on original experimental records, data not published in numerical format. Data adapted from Pi et al., 2020, with permission.

**Supplementary Table S2** Primers used for qRT‑-PCR validation

| **Gene Name** | **Bv_ID** | **Forward primer** | **Reverse primer** |
| --- | --- | --- | --- |
| *BvFVE1* | Bv7_176780_srgi | CATTGCAGATATGGCGCATTA | ACATGATATTTGCATCCTTCCAAGT |
| *BvRNP1* | Bv3_056190_iakn | GTTCATGCGTGTGAGGGAGA | GCAGCCCCCTTAGGCTGT |
| *BvHDT1* | Bv9_211080_qkgc | GACTCTGATGAGGATATGCAAAGTT | CGCCCTTCTTCCCATCTGTT |
| MSTRG.29583.1 | — | GAGAGCGGATGAGTGGTAGC | CGCTCTTGGTGTTGCTCGT |
| MSTRG.9690.1 | — | AGCAATCGGGTCAAACAGGT | GGTAAACAGGTCAGAACCTATGAA |
| MSTRG.38394.1 | — | AACTGGTGGAAGCCGAACAA | GTTCACAAGACCACAACTCAACCA |
| *BvGAPDH* | Bv5_110740_ygnn | GCTTTGAACGACCACTTCGC | ACGCCGAGAGCAACTTGAAC |

# 2 Supplementary Figures

**Supplementary Figure S1** The transcriptional regulatory network of vernalization-related DE-mRNA encoding nuclear proteins. (163 regulatory pairs between 39 transcription factors and 125 nuclear‑encoded DE‑mRNAs).

**Supplementary** **Figure S2** The potential DE-miRNA regulating vernalization-related protein modification enzymes. (A) The network between DE-miRNA and DE-mRNA encoding protein modification enzymes. (B) The expression pattern of DE-miRNA.

**Supplementary Figure S3** The potential DE-miRNA regulating vernalization-related transcription factor. (A) The network between DE-miRNA and DE-mRNA encoding transcription factor. (B) The expression pattern of DE-miRNA.
